# Special Issue “Antimicrobial Biomaterials: Recent Progress”

**DOI:** 10.3390/ijms25137153

**Published:** 2024-06-28

**Authors:** Helena P. Felgueiras

**Affiliations:** Centre for Textile Science and Technology (2C2T), University of Minho, Campus de Azurém, 4800-058 Guimarães, Portugal; helena.felgueiras@2c2t.uminho.pt

Biomaterials have demonstrated their ability to serve as effective drug delivery platforms, enabling targeted and localized administration of therapeutic agents. The development of biomaterial-based drug delivery systems has been particularly beneficial in the management of various medical conditions [1,2]. In the field of cancer treatment, biomaterial-based nanoparticles and liposomes can precisely deliver chemotherapeutic agents to cancerous cells, thus reducing the impact on healthy tissues and minimizing side effects [3]. In the treatment of cardiovascular diseases, biomaterial-coated drug-eluting stents have been shown to release pharmaceutical agents locally, reducing the risk of restenosis [4]. Moreover, biomaterials have played a crucial role in overcoming the blood–brain barrier, allowing for neurodegenerative diseases like Alzheimer’s and Parkinson’s to be treated by target-directed drug delivery vehicles [5]. Biomaterial scaffolds in wound healing have also demonstrated the ability to facilitate cell growth while fighting infections [6,7].

The versatile nature of biomaterials continues to drive advancements in drug delivery, paving the way for more effective and patient-centered healthcare solutions [8,9]. Innovations in nanotechnology and gene therapy, for instance, have further enhanced personalized drug delivery strategies based on individual genetic profiles and disease characteristics [10,11]. Real-time monitoring and feedback systems can be engineered to dynamically adjust drug release rates, ensuring that patients receive precise doses when needed [12].

Biomaterials play a crucial role in the development of implantable devices and drug delivery platforms, significantly impacting patients’ quality of life. The versatility of biomaterials allows for the engineering of a diverse array of devices and scaffolding systems, each tailored to specific patient demands. However, the long-term use of these implantable devices can be threatened by the adhesion and proliferation of microorganisms, leading to the formation of biofilms or fibrotic responses that can become highly cytotoxic and endanger the patients’ quality of life [13]. Pathogenic microorganisms may cause local infection and, consequently, implant failure; further, they can hinder the delivery of therapeutic molecules by specialized delivery platforms, rendering them ineffective. Many alternatives have been proposed over the years to prevent such events, including the use of antiseptics and antibiotics or the physical modification of the biomaterial’s surface, with the incorporation of bioactive agents of interest. From specialized polymers and functional groups to silver and, more recently, antimicrobial peptides and natural extracts, different functionalization/modification techniques have been employed in this fight against pathogenic invaders [8,14]. In fact, the interest in the loading of natural and synthetic antimicrobial agents into different inorganic, lipid, and polymeric-based nanomaterials has been reignited [15]. Not only that, but patients with specific infections, such as ventriculostomy-induced infections, have benefitted from the impregnation and decoration of biomaterials with antimicrobial agents [16]. Silver–porous aluminum oxide nanocomposites, for instance can be synthesized using microwave irradiation. The microwave treatment helps incorporate the silver nanoparticles into the porous alumina matrix, creating stable heterojunctions with improved antiseptic and bactericidal properties [17]. Additionally, antibiotics combined with nanomaterials have been shown to surpass the problems with multi-drug resistance, turning obsolete antibiotic-based therapies into new and relevant approaches for fighting infections [18]. In another strategy, a low-temperature atmospheric argon plasma brush combined with antibiotic-like agents was used for oral bacterial deactivation [19]. More recently, antimicrobial peptide-based hydrogels were explored as alternative agents, as they are increasingly examined for their potent antimicrobial properties and practical applications, such as in wound healing [20].

The emergence of drug-resistant tuberculosis is a significant global health issue, with *Mycobacterium tuberculosis* being responsible for drug-resistant cases. Effective detection of this bacterium is a great challenge. Researchers have engineered super-selective primer-based real-time PCR assays, through which efficient diagnoses of hetero-resistant tuberculosis in clinical specimens can be accomplished, while contributing to the understanding of the bacterium’s drug resistance mechanisms [21]. The challenge to discover new antimycobacterial agents against this bacterium has forced researchers to explore new classes of antimicrobial compounds, including repurposed drugs like gatifloxacin, moxifloxacin, and various natural compounds with anti-tuberculosis properties [22]. Aside from this, other infections have been the target of research for new antimicrobial formulations, some of which are engineered from natural resources, such as quaternized soy protein isolates, which have shown significant antibacterial properties and superior water solubility [23]. Biosynthesized silver chloride nanoparticles from the leaf extract of *Carduus crispus* have also been examined for their antibacterial efficacy, showing great potency against Gram-positive and Gram-negative bacteria [24].

In the food industry, the preservation of fruits and vegetables to prevent propagation of infections is a great challenge. However, some advances are being made, namely the application of compounds based on chitosan, phosphoric acid plus micronutrients, and sweet orange essential oil to coat the aliments and reduce decay. These coatings increase the antioxidant and flavonoid content at harvest and reduce the use of chemical–synthetic fungicides used for preservation [25]. Chitosan beads immobilized with cellobiose dehydrogenase have also been proposed for packaging in the food industry to improve antioxidant, antimicrobial, and cytotoxic properties [26]. Likewise, chitosan can be used for nanoencapsulation of different antimicrobial agents to improve the biological activity of packaging materials against various pathogens [27].
